# The effect of landscape structure on the distribution of brown hare *Lepus europaeus* in farmlands of Germany and Poland

**DOI:** 10.1007/s13364-012-0091-z

**Published:** 2012-08-18

**Authors:** R. Kamieniarz, U. Voigt, M. Panek, E. Strauss, H. Niewęgłowski

**Affiliations:** 1 Polish Hunting Association, Research Station, ul. Sokolnicza 12, 64-020 Czempiń, Poland; 2 University of Veterinary Medicine Hannover—Foundation, Institute for Terrestrial and Aquatic Wildlife Research, Bischofsholer Damm 15, 30173 Hannover, Germany

**Keywords:** Brown hare, Density, Crop fields, Habitat, Spatial distribution

## Abstract

Habitat management should be an important part of the brown hare (*Lepus europaeus*) conservation, but the habitat requirements of this species are not fully recognised. The aim of our research was to estimate these requirements by analysing the effect of various agricultural landscape structure features on the distribution of hares in five agricultural areas in Germany and Poland. The local density of hares was assessed in the spring and autumn of 2006 by using the method of spotlight–strip counts on 9–15 subareas in each research region. The structure of agricultural landscape has been described for each subarea: the share of grain, other crops and grasses as well as the density of crop edges and uncultivated places with wild vegetation. The density of hares was considerably higher in Germany than in Poland (18.8–48.4 vs. 4.1–9.5 indiv./km^2^). The hare density was positively correlated with non-grain crops in an area, with crop edges in two areas and with wild vegetation without trees in two areas, and negatively correlated with grassfields in two areas. The occurrence of wild vegetation without trees affected the hare density only in the study areas, where this habitat was relatively rare (<3 km/km^2^). It was suggested that proper projects aimed at habitat management for brown hares should be elastic, i.e. the projects should be modified depending on the structure of local landscapes. Moreover, the protection and creation of structures with wild vegetation among cropland seem to be considerable methods of brown hare or generally wildlife conservation; therefore, such measures should be an important part of agro-environmental packages.

## Introduction

The number of brown hares (*Lepus europaeus*) in Central Europe, including the territory of Germany and Poland, decreased significantly within the last two decades of the 20th century (Strauss and Pohlmeyer 2001; Kamieniarz and Panek 2008). The intensification of agriculture, i.e. the increase of field sizes, the homogenisation of crop structure and the decrease of unmanaged areas with wild vegetation, is considered one of the crucial causes of this phenomenon (Tapper and Barnes 1986; Panek and Kamieniarz 1999; Smith et al. 2005). It may lead to the limitation of food resources and especially the availability of vegetation containing sufficient amount of specific nutrients, as many plant species preferred by the brown hare grow at the edges of crop fields and wastelands (Endler and Jezierski 1995). In contrast, the better food resources result in more fertile females and better condition of young hares as well as limiting the proportion of females without litter in a given year (Hackländer et al. 2002). In areas of low habitat diversity, Frylestam (1980) found lower body weights, higher mortality rates and smaller litters of brown hare. Moreover, the structure of agricultural landscape may determine the rate of predation on hares. For instance, in Poland, hares were less frequently caught by their main predators, i.e. red foxes (*Vulpes vulpes*), in more diversified habitats than in homogeneous ones, especially in the areas with low density of hares (Panek 2009). The interactions between the agricultural landscape structure and predation risk may mean that habitat deterioration, among other negative effects, will also cause the increase of predation pressure on farmland animals (Evans 2004; Smith et al. 2005). Therefore, the occurrence of hares in agricultural areas is, to a large extent, conditioned by the direction of changes in cultivation by humans (Smith et al. 2005).

The changes in agricultural landscape also affect the abundance of other species inhabiting open areas, e.g. grey partridges (Potts 1986). That is why solutions are sought, which are not only more beneficial economically, but also more environmentally friendly, resulting in discussions to change the European Union Common Agricultural Policy (Münchhausen and Börner 2003). Among others, the agro-environmental programs serve the purpose of balanced development and protection of biodiversity in agricultural landscapes. The packages, which have been implemented so far and more often comprised sowing various periodical crop plants on selected parts of arable land, did not always result in positive effects expected by naturalists (Ringler and Steidl 2004). Therefore, guidelines for the modification or creation of new packages are necessary (Oppermann et al. 2009; Thomas et al. 2009). Consequently, the relationships between the changes in farmland areas and the occurrence of animals preferring that type of habitat should be known better.

Smith et al. (2005) analysed 77 publications on the relation between the abundance of brown hares and their habitat. They found positive associations with arable land, various crops and fallow land and negative with monocultures. However, individual studies often showed opposite results, for instance in about half of them, a negative impact of the growth in the field size on the hare density was mentioned, but in the second half of publications, the same factor was considered neutral, and in one, it was positive. Consequently, the meta-analysis did not provide evidence that the field size affects hare numbers (Smith et al. 2005). Such differences in individual studies could be the result of different spatial scales, applied methods or definitions of variables describing the habitat structure. But first of all, these differences could be related to different ranges of habitat variables occurring in individual study areas. For example, the field size may not be important in regions with only small fields, but it may affect local hare abundance in regions with partially large fields. This aspect was poorly studied previously.

The aim of this research was to find out habitat requirements of brown hares by analysing the effect of various agricultural landscape structure features on the distribution of these animals, i.e. their local density, within five study areas in Central Europe. These areas differed in agricultural landscape structure, and we predicted that hare–habitat relations will differ depending on the range of habitat variables in individual areas.

## Materials and methods

### Study areas

The study was carried out in 2006 in highly productive low-forested agricultural areas. In Germany, the areas were located in the north-west lowlands within 15–25 km from Hannover and marked as G1 (N 52°20′, E 09°33′), G2 (N 52°14′, E 10°05′) and G3 (N 52°10′, E 10°36′). In Poland, “P1” area was located in west Poland, 30 km south from Poznań near Czempiń (N 52°08′, E 16°45′). “P2” area was located in central Poland, 60 km north from Łódź, around Krośniewice (N 52°15′, E 19°10′). The areas were chosen to represent typical agricultural systems in western Germany and Poland, for example landscapes with domination of previous large state farms (P1) and with only small family farms (P2), respectively, in western and central-eastern parts the second country. In all study areas, agricultural lands dominated and covered on average 90–97 % of the area. The remaining part constituted non-cultivated places usually overgrown with wild permanent vegetation (3–10 %).

### Data collection

**Fig. 1:**
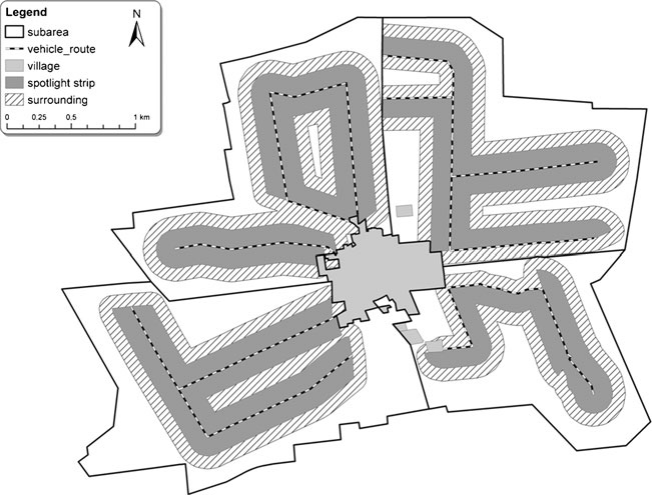
Distribution of subareas, spotlight count strips and surrounding zones within a study area

In each study area, from 9 to 15, subareas of a size of 2–5 km^2^ were determined. In these subareas, the density of hares was estimated as well as the structure of agricultural landscape was described. Hare densities were assessed in spring and autumn of 2006 by spotlight–strip counting, a standardised method described by Bartel et al. (2003). The animals were counted during night hours using spotlight (12 V, 55 W) from a slowly moving vehicle on the strip of open land parallel to the vehicle route within a distance of 150 m. The counting strips were distributed in subareas along the existing local and field roads (Fig. 1). The surveyed surface in the subareas reached from 0.3 to 2.0 km^2^, giving total surfaces from 7.7 to 12.4 km^2^ in the five study areas. The spotlight counts were carried out in spring from the beginning of March until the end of April, when observation was possible due to low vegetation. In addition, the counts were conducted in autumn from October to November after the majority of crop harvest was completed but before the initiation of hunting. At least two counts were done in each season. If the number of counted hares differed more than 25 % from the average of two counts, a third count was carried out (Bartel et al. 2003). Since our subareas for hare counts were rather small, we assumed that the observed differences in local hare density described differences in their distribution (which probably depends considerably on daily and seasonal movements) rather than in the population density level (which is mainly a result of reproduction and survival).

The landscape structure was mapped within the counting strips and surrounding zones which were defined as a 100-m buffer on both sides around the counting strips (Fig. 1). Depending on the length of the counting strips, the mapping areas for landscape structure ranged from 0.4 to 3.2 km² for the subareas and from 13.5 to 20.8 km² in the five study areas. The measurements were obtained using a GPS receiver or rangefinder and GIS, respectively. Next, the results were put on the map using a scale of 1:10,000. Based on the maps, the area or length of landscape structures was calculated for each subarea and expressed in area percentage or as density of line and strip structures (kilometre per square kilometre). The following variables were used in the analysis:

- grains, percent;
- other crops, percent: mainly oil-seed rape, alfalfa and clover, mustard and other green cover crops, as well as stubbles and shallowly ploughed stubbles, where self-sown crops and wild plants occurred (cover >10 %);
- grasses, percent: meadows and pastures;
- crop edges, kilometre per square kilometre: direct borders between different crops (or crop/plough borders) without strips of wild vegetation or with such strips up to 0.5 m in width;
- wild vegetation without trees, kilometre per square kilometre: linear structures of more than 0.5 m in width (total length) and patches (length of all borders), e.g. drainage ditches, roadsides, hedgerows, border strips, mid-field clumps typically up to several hectares and fallow fields covered with spontaneous grasses and forbs, and sometimes also shrubs;
- wild vegetation with trees, kilometre per square kilometre: as in the previous variable, but additionally with rows or clumps of trees;
- building edges, kilometre per square kilometre, i.e. borders of build-up areas, for example farms (such areas were excluded from the subareas)—we added this variable to remove potential effects of buildings on hare distribution.

### Statistical analyses

The density of hares in respective areas and seasons was compared by analysis of variance. The relationships between hare density and landscape structure were determined using covariance analysis. Partial correlation coefficients (*r*_p_) were estimated. Our data were collected in the same subareas in two seasons. However, in fact, habitat characteristics of our subareas differed between spring and autumn as crop composition changed and, consequently, also the occurrence of crop edges was modified. Moreover, reproduction in hare populations took place between the two periods of data collection, thus changes in the distribution of hares were probable. Therefore, the data from each subarea and season were assumed as independent samples. However, the factor “season” was used in analyses to remove the effect of potential differences in the level of hare density. Accordingly, an analysis of covariance was carried out for each study area with simultaneous use of all landscape structure variables and season, i.e. final linear model incorporating all variables was calculated. Finally, partial correlation coefficients obtained from the above model for the most important variables describing landscape structure (crop edges, wild vegetation without trees, wild vegetation with trees) were correlated against the mean values of these variables in each study area. So, we checked if the magnitude of relation between hare abundance and a given landscape variable changed with the level of occurrence of this variable in individual areas. STATISTICA software was used in all analyses.

## Results

The average hare densities in the five study areas in Germany and Poland in 2006 varied considerably and ranged from 5.5 to 42.2 individuals/km^2^ in spring and from 4.1 to 48.4 individuals/km^2^ in autumn. The densities differed significantly between areas, but no differences were found between seasons (two-way analysis of variance: area—*F* = 72.121, *p* < 0.001; season—*F* = 0.037, *p* = 0.8; area*season—*F* = 0.932, *p* = 0.4). The average densities were considerably higher in Germany than in Poland (Table 1). The means and ranges for the variables of landscape structure in individual study areas during spring and autumn are shown in Table 2.

**Table 1:** Brown hare density (individuals per square kilometre) in five study areas in Germany (G) and Poland (P) each for spring and autumn 2006

Study area (number of subareas)	Spring			Autumn		
	Mean	SD	Range	Mean	SD	Range
G1 (11)	22.2	8.6	12.0–36.3	19.0	10.2	7.5–45.4
G2 (15)	42.2	11.4	20.5–69.1	48.4	16.5	11.5–67.5
G3 (9)	20.2	12.2	6.6–42.3	18.8	11.5	3.9–37.7
P1 (12)	5.5	3.0	1.8–10.7	4.1	2.8	1.0–9.5
P2 (12)	8.0	3.5	3.1–13.8	9.5	5.8	2.6–23.1

**Table 2:** Variables describing the landscape structure (mean and ±SD in parentheses), i.e. area percentages or densities of linear structures (kilometre per square kilometre), in five study areas in Germany (G) and Poland (P) during counting periods of hares in 2006 (*s* spring, *a* autumn)

Variable	Season	Study area				
		G1	G2	G3	P1	P2
Grains (%)	s	59.4 (±8.6)	53.5 (±11.7)	59.0 (±14.4)	45.2 (±14.2)	50.3 (±13.0)
	a	43.5 (±11.7)	37.8 (±15.8)	46.2 (±15.8)	39.4 (±12.1)	35.4 (±17.0)
Other crops (%)	s	12.4 (±8.2)	7.7 (±9.4)	19.9 (±14.5)	13.9 (±9.0)	7.6 (±7.1)
	a	30.6 (±12.4)	23.8 (±12.5)	36.5 (±13.0)	19.0 (±10.0)	16.8 (±17.2)
Grasses (%)	s	2.2 (±2.5)	4.8 (±6.5)	0.9 (±1.2)	1.7 (±3.3)	5.1 (±3.5)
	a	2.5 (±3.1)	4.5 (±6.6)	1.0 (±1.2)	1.7 (±3.3)	5.8 (±4.4)
Crop borders (km/km^2^)	s	1.5 (±1.1)	2.4 (±1.4)	2.0 (±0.9)	4.4 (±4.1)	25.5 (±11.6)
	a	1.4 (±0.9)	3.0 (±1.7)	2.3 (±1.0)	4.5 (±3.7)	24.6 (±11.2)
Wild vegetation without trees (km/km^2^)	s	4.6 (±2.3)	3.7 (±1.1)	2.4 (±1.1)	1.7 (±0.9)	6.8 (±2.4)
	a	4.5 (±2.4)	3.9 (±1.3)	2.3 (±1.1)	1.7 (±0.9)	6.7 (±2.5)
Wild vegetation with trees (km/km^2^)	s	4.6 (±2.4)	2.8 (±1.3)	2.6 (±1.8)	1.8 (±0.8)	1.9 (±1.3)
	a	4.7 (±2.5)	2.8 (±1.3)	2.6 (±1.8)	1.8 (±0.8)	1.9 (±1.3)
Building edges (km/km^2^)	s	0.7 (±0.6)	0.6 (±0.5)	0.4 (±0.2)	0.4 (±0.4)	1.1 (±0.9)
	a	0.9 (±1.1)	0.6 (±0.5)	0.4 (±0.2)	0.4 (±0.4)	1.1 (±0.9)

An analysis of covariance for hare density in relation to all landscape structure variables and season was carried out for each study area (Table 3). In two areas, G1 and P2, none of the obtained partial correlation coefficients was significant. However, in G2, the hare density was negatively correlated with the share of grasses and positively correlated with the occurrence of crop borders. In G3, only the positive partial correlation coefficient for the occurrence of wild vegetation without trees turned out to be significant. In P1, significant positive effects were found of other crops, crop borders and wild vegetation without trees; however, grasses and the occurrence of building borders had a negative effect. The impact of season turned out to be significant only in the latter area, which may indicate seasonal differences in the relation between the hare density and landscape structures.

**Table 3:** Partial correlation coefficients between hare density and all variables of agricultural landscape structure in five study areas in Germany (G) and Poland (P) in 2006

	Study area (df)				
Variable	G1 (1, 13)	G2 (1, 21)	G3 (1, 9)	P1 (1, 15)	P2 (1, 15)
	Partial correlation coefficients (*r*_p_)				
Grains (%)	−0.002 ns	0.059 ns	−0.080 ns	0.182 ns	0.238 ns
Other crops (%)	0.407 ns	0.047 ns	−0.301 ns	0.668**	−0.116 ns
Grasses (%)	0.323 ns	−0.513*	0.237 ns	−0.520*	0.121 ns
Crop borders (km/km^2^)	−0.119 ns	0.441*	0.386 ns	0.655**	0.302 ns
Wild vegetation without trees (km/km^2^)	0.227 ns	−0.193 ns	0.773**	0.517*	−0.390 ns
Wild vegetation with trees (km/km^2^)	−0.194 ns	0.253 ns	0.424 ns	0.330 ns	0.026 ns
Building edges (km/km^2^)	−0.472 ns	0.101 ns	−0.089 ns	−0.673**	0.392 ns
Season	*F* = 2.490 ns	*F* = 0.126 ns	*F* = 0.205 ns	*F* = 6.073*	*F* = 2.519 ns
*R* ^2^ (%)	46.0 ns	48.5*	81.2*	67.7*	46.6 ns

Analyses of covariance were carried out with simultaneous use of all structure and season variables for a given area (*p < 0.05, **p < 0.01, *ns* not significant; *df* degree of freedom; *R* ^2^ coefficient of determination)

**Fig. 2:**
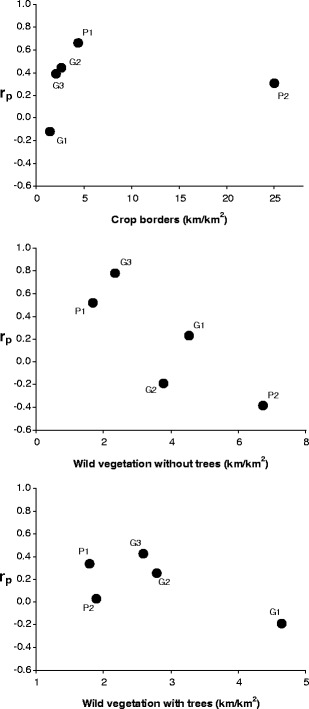
Partial correlation coefficients (*r*_p_) between hare density and three variables describing landscape structure in relation to the average value of these variables in five study areas in Germany (G) and Poland (P) in 2006 (crop borders: *r* = 0.036, df = 3, *p* = 0.95; wild vegetation without trees: *r* = −0.839, df = 3, *p* = 0.08; wild vegetation with trees: *r* = −0.658, df = 3, *p* = 0.2)

In order to check whether the character or the power of the relationships between the hare density and specific landscape structure features depends on the level of their occurrence in a given area, partial correlation coefficients (Table 3) for three basic variables describing the landscape structure (crop borders, wild vegetation without trees and wild vegetation with trees) were compared with the average values of these variables in individual areas. In the case of wild vegetation without trees, the correlation turned out to be high and close to being significant (Fig. 2). The relation between hare density and the occurrence of wild vegetation without trees changed from being positive (where it amounted to less than 3 km/km^2^) towards a negative one depending on the amount of such vegetation present in the area. For the two other variables, the coefficients of correlation with hare density were far from significance (Fig. 2).

## Discussion

Our research partially confirms the findings of recent synthesis concerning hare–habitat relations carried out by Smith et al. (2005). For example, they showed that the occurrence of various crops has a positive effect on hare abundance. In our case, the share of non-grain crops positively influenced hare density only in one of the study areas. This association was probably related to the importance of non-grain crops as food source and cover for hares (Smith et al. 2005). A similar phenomenon might be expected in the case of grain. It was found that grain fields were selected by brown hares during feeding because grain constitutes an important element of their diet, especially during the period when these plants are low and have proper nutritious values, i.e. from late autumn to early spring (Tapper and Barnes 1986; Smith et al. 2004; Reichlin et al. 2006). Consequently, grain was often positively associated with hare abundance (Smith et al. 2005). However, the spatial variation of the occurrence of grain had no significant effect on the distribution of hares in all our study areas. In these areas, grain was present in all subareas and covered on average about a half of agricultural lands. Thus, grain was commonly available for hares, and probably for this reason, their occurrence did not considerably limit their distribution, even if grain fields were often used by them as feeding places. Moreover, as the proportion of grain in our study areas reached high values in some subareas, they sometimes occurred practically as monocultures. In this case, the impact of grain may even be negative. In monocultures, the availability of food decreases periodically because the crops grow and mature at the same time, becoming useless for hares (Frylestam 1980, 1986). Consequently, if grain covers large areas, they limit the access to other sources of food (Schmidt et al. 2004). It may be another reason for the lack of positive effect of grain on local hare density in this study. On the other hand, it is probable that the occurrence of grains and other crops limits utility of agricultural land for hares and, consequently, affects their distribution also in regions with very small proportion of green plant.

The sources of diverse and high caloric food for brown hares also included grassland plants as grasses and herbs constitute the main part of their diet (Homolka 1987; Chapuis 1990). Hares prefer short grasses, and their feeding intensity decreased in places covered with tall-growing plants (Kuijper et al. 2008). Due to periodical rejuvenation of grassland plants by mowing or livestock grazing, they seem more attractive to hares than grain. Therefore, grass fields were sometimes indicated as the most important element of hare habitat (Prigioni and Pelizza 1992), affecting hare density positively especially in spring (Meriggi and Alieri 1989). In Western Germany, the highest density of hares was recorded in intensively used agricultural landscapes, mainly in croplands; however, high densities were also found in grasslands used as pastures (Strauss et al. 2008). However, according to the review by Smith et al. (2005), in pastoral habitats, hare density was usually lower than in arable land. Moreover, in areas where arable land dominates, the presence of grass fields sometimes negatively affected hare density, as it was found in France by Pepin (1986, 1987) and Marboutin and Aebischer (1996) as well as in two of our study areas. The ambiguous effect of pastures seems to result from the different impact of grazing livestock on the content and structure of such plant communities (Smith et al. 2004). The negative impact of grass patches among arable land on hare distribution may also be the effect of predator avoidance. Grass fields may be frequented by red foxes due to the abundance of small rodents, one of the main ingredients of foxes' diet (Goszczyński 1995; Russell and Storch 2004). Therefore, the effect of grassland on brown hare distribution and density seems to be dependent on the way of its management and use by other species as well as on the occurrence of other feeding habitats.

So far, published studies devoted to the relation between the occurrence of hares and agricultural landscape structure in about a half of the investigations indicated a positive impact of small field sizes on hare density (Smith et al. 2005). Similarly, in our research, the occurrence of crop borders, depending on field sizes, positively affected the occurrence of hares in two of five study areas. In Poland, it was previously found that brown hares clearly preferred edges of crop fields, especially in regions with highly monotonous crops and relatively large fields (Lewandowski and Nowakowski 1993 *).* Therefore, it can be expected that the positive effect of crop borders does not occur in areas with very small fields (as in P2). On the other hand, such effect seems also unlikely in areas where direct borders between crops (i.e. without strips of permanent vegetation) are very rare; thus, they are practically unavailable for hares.

Another feature of agricultural landscape structure, which positively affected the local density of hares in this study, was wild vegetation growing on non-cultivated places among arable fields. Fallow land with permanent cover undoubtedly provides hares with food and cover throughout the year. According to Reichlin et al. (2006), wild plants constitute an important diet ingredient of hares. Therefore, this type of habitat is positively associated with hare abundance (Smith et al. 2005). However, the occurrence of places with wild vegetation without trees significantly affected hare distribution only in our study areas with low amounts of such places where hares have probably locally limited access to them, but non-significant correlations were found in the areas where wild vegetation was abundant, thus commonly available for hares. Therefore, our results seem to show that fallow land with wild herbaceous plants is very important for brown hares, but they do not require its high proportion among agricultural land.

Moreover, no significant impact on local hare density was also found in the case of wild vegetation with trees, although it included herbaceous plants that may serve as food and shelter for these animals. In Polish agricultural landscapes, the lack of positive effect or even the avoidance of places with trees and forest edges as well as lower densities in areas with their common occurrence was formerly observed in both hares and partridges (Panek and Kamieniarz 1998, 1999). This was probably due to the preference of trees by some predators. Only if there is a very small share of places with trees in the vast open agricultural areas, their impact on the occurrence of hares may be positive (Schneider and Maar 1997; Vaughan et al. 2003). Moreover, the importance of places with trees may depend on their character; for example, it was found in Italy that hares preferred mature poplar plantations but avoided natural woods (Meriggi and Verri 1990; Cardarelli et al. 2011).

We did not find agricultural landscape features that affected the distribution of hares in all study areas. Some features were significant in a given area but insignificant in others. There are several reasons why this is the case. For example, even though the same definitions of landscape variables were used in all areas, the character of these variables may, in fact, differ between areas (e.g. various kinds of grasslands). The given feature may be rare in some areas but present in excess in others (e.g. wild vegetation without trees). In addition, the effect of particular variables on hare distribution may depend on the occurrence of other variables (e.g. trees in diverse and very simplified farmlands). Therefore, proper national and even regional projects aimed at diversification of farmland habitats for the conservation of brown hares should be flexible, i.e. the projects should be modified depending on specific features of local landscapes.

Overall, it may be emphasised that although our findings were not uniform, generally, brown hares often responded positively to some features related to the diversity of agricultural landscape, particularly to abundant occurrence of places with wild treeless vegetation and crop borders. Unfortunately, agricultural landscapes are subject to negative changes resulting from agriculture intensification, especially expressed in the increase of field sizes and deterioration of fallow land as well as grain monocultures. According to our results, proper habitat management for hares should include not only sowing additional plots of periodical crop plants, but first of all, protection of permanently uncultivated pieces of land with wild grasses and dicotyledonous herbs and creation of such new places in areas with their rare occurrence. Therefore, the points related to the protection and creation of structures with wild vegetation among agricultural landscapes should be an important part of the agro-environmental packages and should be often used for the conservation of the brown hare and generally wildlife in farmland areas.
